# REPLY FROM DR. STEPHEN M. SAGAR

**Published:** 2007-12

**Authors:** Stephen M. Sagar

**Affiliations:** Juravinski Cancer Centre, 699 Concession Street, Hamilton, Ontario L8V 5C2., E-mail: stephen.sagar@hrcc.on.ca

I agree with Dr. Milgrom that some interesting phenomena have been discovered in the supramolecular modification of water structure and also in molecular semiotics. However, the link to clinical effects has not been established. My romantic rationality yearns for these effects to have clinical application. What clinician or patient would not want negligible toxicity?

Unfortunately, material science data is being fitted retrospectively to a paradigm of homeopathy that has not been proven to result in clinical effects (over and above placebo) in the real world (inductive reasoning). Therefore my logical rationality cannot condone this. Misinterpretation is pseudoscience.

The practice of homeopathy encourages expectation and the so-called placebo effect—and that’s fine. However, it is nonsense to utilize expensive homeopathic preparations without the proof that the compound itself has an effect in addition to the therapeutic relationship or, indeed, the packaging. I support individualization of therapies, but this is no excuse for not undertaking a randomized controlled trial, because randomization can be performed after the individualization decision.

Regarding funding, if the many companies that profit from the sale of expensive homeopathic remedies were to contribute to research, then the truth would be revealed. Many of the over-the-counter remedies do not individualize, but claim a blanket cure for all.

Note that in my editorial, I did not use the metaphor “a spoonful of sugar.” A “teaspoon of honey” leaves open the option that there may be other substances besides sugar that can result in a therapeutic effect. (Honey contains more than sugars.) However, it is the responsibility of the scientist to authenticate these substances and to prove within the clinical scenario that they contribute more than expectation or the placebo effect.

I am sure that Hippocrates is somersaulting in his grave, celebrating the many effective therapies that allopathic medicine has developed for our patients with cancer. As a humane clinician and a scientist, I support any intervention that benefits our patients, but I do not endorse recommendations that are misleading and not supported by reasonable and rational evidence. If homeopathy is to advance, it requires deductive reasoning and suitable clinical trials that exclude the powerful effects of expectation alone.

Sincerely,

